# The Effect of Ice-Binding Protein from *Leucosporidium* sp. AY30 (LeIBP) on the Physicochemical Quality and Microstructure of Largemouth Bass During Freeze–Thaw Cycles

**DOI:** 10.3390/foods13244038

**Published:** 2024-12-13

**Authors:** Junde Ren, Maninder Meenu, Lihui Hu, Tao Song, Ying Liu, Hosahalli S. Ramaswamy, Yong Yu

**Affiliations:** 1 College of Biosystems Engineering and Food Science, Zhejiang University, Hangzhou 310058, China; 22213008@zju.edu.cn (J.R.); meenu@zju.edu.cn (M.M.); xytsmf474@163.com (L.H.); 22313014@zju.edu.cn (T.S.); liuyingzju@zju.edu.cn (Y.L.); 2 Key Laboratory of Equipment and Informatization in Environment Controlled Agriculture, Ministry of Agriculture, Hangzhou 310058, China; 3 Department of Food Science and Agricultural Chemistry, McGill University, 21111 Lakeshore Road, Sainte-Anne-de-Bellevue, QC H9X 3V9, Canada; hosahalli.ramaswamy@mcgill.ca

**Keywords:** ice-binding protein, *Leucosporidium* sp. AY30, largemouth bass, freeze–thaw cycle, microstructure

## Abstract

This study investigated the effect of various concentrations (0.01%, 0.05%, 0.1%, 0.2%, 0.5%) of ice-binding protein from *Leucosporidium* sp. AY30 (LeIBP) on the freezing efficiency, microstructure, and physicochemical quality of largemouth bass during freeze–thaw cycles and demonstrated the optimal addition conditions of LeIBP. This study found that LeIBP could effectively lower the freezing point of fish without altering the phase transition time significantly. LeIBP can significantly reduce the cross-sectional area and diameter of ice crystals and inhibit recrystallization. LeIBP was found to maintain the stability of protein secondary structure and prevented protein denaturation by increasing the proportion of α-helix. The inclusion of LeIBP retained the water-holding capacity of fish effectively. Furthermore, LeIBP treatment could partially prevent the degradation of fish meat texture. The lightness and whiteness values of fish treated with LeIBP were increased, while the redness and yellowness values were decreased. At the end of freeze–thaw cycle, the LeIBP-treated group presented pH values similar to fresh fish. Overall, 0.05% LeIBP was observed to be the most effective concentration to inhibit ice crystal growth, thereby maintaining the quality of the fish.

## 1. Introduction

Largemouth bass (*Micropterus salmoides*) is an important aquaculture and fishing species renowned for its high economic value and exquisite flavor. Largemouth bass contains about 5% lipid content and a significant amount (>50%) of polyunsaturated fatty acid [1]. As per the 2023 China Fisheries Statistical Yearbook, the yield of freshwater perch aquaculture in 2022 soared to 802,486 tons, reflecting a notable increase of 14.30% since 2021 [2]. With the surge in the production of freshwater perch, in addition to fresh food supply, a large number of freshwater perch need to be frozen and preserved for circulation to more areas and the development of related products, such as prepared dishes, in order to meet the needs of people’s high-quality protein supplements.

Air-freezing emerges as an extensively utilized freezing technology which employs air as a freezing medium. However, the low thermal conductivity of food and poor heat transfer coefficient of air result in a slow freezing rate of aquatic products. This in turn leads to the formation of large ice crystals and significant damage to cells and results in poor-quality aquatic products [3]. Concurrently, owing to the inadequacies in cold chain technology, disruptions in the cold chain are frequently observed during the processing, storage, transportation, and distribution of aquatic products. The resultant temperature fluctuations cause repeated freezing and thawing of aquatic products [4,5,6]. During multiple freeze–thaw cycles, the phenomenon of recrystallization occurs, which causes irreversible damage to cells, consequently resulting in nutrient loss and poor-quality aquatic products. This results in significant food and economic loss. Consequently, effective control of ice crystal growth during freeze–thaw cycles is a crucial issue that require effective solution during the handling, conveyance, and preservation of frozen comestibles.

As a novel ice-modifying substance, antifreeze proteins (AFPs) control the crystallization of ice, thereby enhancing the quality of frozen comestibles, and exhibit significant potential in the frozen food industry [7]. When AFPs adhere to the surfaces of ice crystals, it results in the formation of a convex surface. Based on the thermodynamic principles, the curvature of the ice surface makes it more challenging for water molecules to adhere, leading to a decrease in the freezing point and a significant reduction in the growth rate of ice crystals surrounding the AFPs [8]. The ice-binding surfaces of AFP molecules and ice crystals are connected by hydrogen bonds, and ice crystals are separated from the water by a hydrophobic non-ice-binding surface, which prevents an increase in the size of the ice crystals [9]. AFPs possess the ability to reduce the freezing point without affecting the melting point. AFPs inhibit the growth and recrystallization of ice crystals induced by freeze–thaw cycles by altering the morphology of ice crystals to safeguard cellular integrity, thereby mitigating protein oxidation and tissue deterioration. That, in turn, enhances the quality of frozen foods [10].

AFPs have been reported from a variety of cold-dwelling organisms, including animals, plants, and microorganisms [11]. Recently, the application of AFPs has been extensively explored in various foods such as meat products, desserts, fruits, vegetables, and dough [12,13,14,15]. Nevertheless, the innate production of fish-derived AFPs is low. Approximately 1–4 g of AFPs can be obtained from 1 L of fish blood [16]. Additionally, the generation of fish AFPs requires a series of intricate procedures, including extraction and purification. The low-yield and complex production process results in the high cost of fish AFPs. Due to this, the application of fish-derived AFPs is not feasible at an industrial scale. Therefore, cost-effective, high-yield, and high-purity AFPs can be produced via heterologous expression using prokaryotic or eukaryotic systems [17].

Recently, a novel antifreeze protein (LeIBP) was discovered in an Arctic cryophilic yeast *Leucosporidium* sp. AY30. LeIBP exhibits unique properties such as thermal hysteresis (TH) and recrystallization inhibition (RI). LeIBP hinders the growth of large ice crystals at the expense of small ice crystals, thus enhancing the viability of cells undergoing freezing and thawing processes [18,19]. The gene of LeIBP was transferred into *Aspergillus niger*, generally recognized as safe (GRAS), to enable recombinant expression and facilitate large-scale production, thereby enhancing yield and reducing production cost. This breakthrough paves the way for its extensive utilization in the aquatic processing industry. Nevertheless, the fundamental data and theoretical investigations regarding the modulation of ice crystal formation in aquatic products by LeIBP are scarce.

Thus, the present study was conducted to explore the impact of different concentrations of LeIBP (0.01%, 0.05%, 0.1%, 0.2%, 0.5%) on the freezing efficacy, microstructure (ice crystal morphology, protein secondary structure), and physicochemical characteristics (pH, water holding capacity, texture, color) of largemouth bass during three freeze–thaw cycles. The present study will gather fundamental data for the application of LeIBP in the aquatic processing industry. This study holds significant practical importance, as the outcomes of present investigation will provide technical guidance and a theoretical foundation for the efficient processing and storage of largemouth bass.

## 2. Materials and Methods

### 2.1. Chemicals

LeIBP was provided by Nanjing Bestzyme Bio-engineering, Co., Ltd, Nanjing, China. CarnoyⅡfixative was obtained from Shanghai Yuanye Biotechnology Co., Ltd., Shanghai, China. Anhydrous ethanol was purchased from Hangzhou Hongda Chemical Instrument Co., Ltd., Hangzhou, China. Xylene, hydrochloric acid, differentiation solution, and neutral gum were procured from Sinopharm Chemical Reagent Co., Ltd., Shanghai, China. Hematoxylin stain was obtained from Hangzhou Haoke Biotechnology Co., Ltd., Hangzhou, China. Eosin stain was purchased from Shenzhen Dakewei Medical Technology Co., Ltd., Guangdong, China. Ammonia was procured from Shanghai Macklin Biochemical Co., Ltd., Shanghai, China. All chemicals used in this study were of reagent grade.

### 2.2. Sample Preparation

Live largemouth bass were purchased from a local supermarket in Hangzhou, China. Fish samples were shipped to the lab within half an hour after being gutted and decapitated and after removing bones and skin. These fish samples were cut into small pieces of approximately 30 mm × 30 mm × 15 mm and stored in sealed polyethylene bags at 4 °C. Different concentrations of LeIBP solutions, namely, 0.01%, 0.05%, 0.1%, 0.2%, and 0.5% (*w*/*w*), were prepared, and the fish pieces were impregnated into different concentrations of LeIBP solutions. Then, these fish pieces were vacuum-packed. The groups were designated as the 0.01% LeIBP group, 0.05% LeIBP group, 0.1% LeIBP group, 0.2% LeIBP group, and 0.5% LeIBP group, and samples without any treatment was labeled as the control group. Then, these six groups were stored in a refrigerator at 4 °C for 4 h. After macerating, the fish pieces were frozen in a −25 °C refrigerator for 3 h, then thawed in a 4 °C refrigerator for 24 h. The freeze–thaw cycle was repeated 1, 2, and 3 times according to the above-mentioned freezing and thawing methods, and the cycles were named FT1, FT2, and FT3, respectively. The sample after the first freezing was named FT0.

### 2.3. Freezing Efficiency

The solder joint needle of the K-type thermocouple (KPS-QB-K-1000-CZ, Kepson, Xuzhou, China) was inserted into the center of the fish block. The sample was placed directly in a −25 °C refrigerator for 3 h, and variations in its temperature were recorded once per second using a data logger (34970A, Agilent Technologies GMBH, Waldbronn, Germany). The data were exported to a computer to plot the temperature–time curve. The average temperature of the platform was considered as the freezing point temperature.

### 2.4. Observation of Ice Crystal Morphology

The ice crystal immobilization treatment was modified based on previous studies [20,21]. The CarnoyⅡfixative was also frozen at −25 °C in advance to ensure its temperature was the same as that of the frozen sample. Then, the frozen sample was placed in CarnoyⅡfixative and fixed at −25 °C for 24 h. After fixation, the sample was removed from the fixation solution for dehydration, paraffin embedding, and sectioning. Then, the paraffin sections were dewaxed to water, followed by hematoxylin staining, eosin staining, and dewatering sealing. Finally, all prepared slides were observed under a microscope (Eclipse E100, Nikon, Tokyo, Japan). The Image-Pro Plus 6.0 software was used to measure the cross-sectional area, maximum diameter, average diameter, minimum diameter, and circumference of the ice crystal section. Then, the roundness and elongation of the ice crystal were also calculated, as mentioned below.

Roundness (R) indicates how close the ice crystal is to the circle. The value of roundness lies between 0 and 1, and the closer the roundness value is to 1, the more round the ice crystal cross-section will be. The roundness of the ice crystal cross-section was calculated according to Equation (1).

(1) $$R=\frac{4\pi A}{P^{2}},$$

Elongation (E) is defined as the ratio of the maximum diameter of the ice crystal cross-section to the minimum diameter, indicating the regularity of the ice crystal. The elongation value 1 indicates a square or circular shape of the ice crystal cross-section. The larger the elongation ratio, the more prominent the tensile deformation of the ice crystal will be, and the regularity of the ice crystal will also be poor. The elongation of the ice crystal cross-section was calculated using Equation (2).

(2) $$E=\frac{{Diameter}_{max}}{{Diameter}_{min}},$$

where A is the cross-sectional area of the ice crystal, and P is the cross-sectional perimeter of the ice crystal.

### 2.5. Protein Secondary Structure

The protein secondary structure of the fish sample was explored by employing Raman spectroscopy, as mentioned in a previous study, with slight modifications [22]. Fish samples from each treatment group were thawed 24 h after each freeze–thaw cycle and cut into 1 mm thick samples, which were placed on a Raman spectrometer (LABRAM HR Evolution, HORIBA Jobin Yvon, Palaiseau, France) for scanning. The objective lens was used at 50×, the laser wavelength was 633 nm, and spectral data were collected from 400 to 2000 cm^−1^. The spectral acquisition time was 5 s, and the number of cycles was 2. The Peakfit 4.12 software was employed for baseline correction, deconvolution processing, and second derivative fitting of spectral data. The resultant spectral data were used to determine changes in the secondary structure of fish protein.

### 2.6. Water Holding Capacity

The thawing loss of fish samples was determined according to a previously described method with slight modifications [23]. Each sample was frozen and weighed (W_0_), then thawed in a refrigerator at 4 °C for 24 h and removed from the polyethylene bag to observe weight (W_1_) after drying the surface with a paper towel.

(3) $$Thawing\;loss(\%)=\frac{w_{0}-w_{1}}{w_{0}}\times 100,$$

The cooking loss in the fish samples was assessed based on a method previously mentioned in the literature with slight modifications [24]. After thawing, each sample was weighed (W_1_) and placed in a Ziplock bag, followed by heating in a water bath at 90 °C for 20 min. The samples were allowed to cool at room temperature for 30 min and then removed from the Ziplock bag, wiped to remove surface water, and weighed (W_2_).

(4) $$Cooking\;loss(\%)=\frac{w_{1}-w_{2}}{w_{1}}\times 100,$$

Furthermore, the total loss in the fish samples was also calculated to determine the total loss that occurred in a sample during thawing and cooking.

(5) $$Total\;loss(\%)=\frac{w_{0}-w_{2}}{w_{0}}\times 100,$$

### 2.7. Texture

The textural parameters, namely, hardness, viscosity, cohesiveness, springiness, gumminess, and chewiness, of the fish samples were analyzed according to a previously described method with some modifications [3]. A texture analyzer (CT3 25K, Brookfield, MA, USA) equipped with a 6 mm cylindrical probe (model TA41) and a deformation target value of 60%, a trigger point load of 5 g, and a test speed of 1 mm/s was used for collecting textural data, and the number of cycles was 2.

### 2.8. Color

The color difference among the fish samples was measured with a spectrophotometer (CM-600D, Konica Minolta, Tokyo, Japan). The lightness (L*), redness (a*), and yellowness (b*) values of the samples were measured. The zero correction and whiteboard correction were applied before collecting data. The whiteness of the fish samples was calculated using the following Equation.

(6) $$W=100-\sqrt{{\left(100-L^{*}\right)}^{2}+a^{*2}+b^{*2}}$$

### 2.9. pH

The pH values of the samples were measured according to the previously reported methods with slight modifications [25]. Before measurement, the pH meter was calibrated at pH values of 4.00 and 9.18. Three pieces of fish were taken from each group and crushed by employing a meat grinder. Then, 5 g of minced fish was mixed with 50 mL of deionized water for 1 min using a digital display high-speed dispersing homogenizer (FJ200-SH, Shanghai Specimen Model Factory, Shanghai, China). The resultant homogenate was centrifuged at 5000 rpm for 10 min at room temperature to collect the supernatant. The pH value of the supernatant was measured with a pH meter (PHS-25, Shanghai Yidian Scientific Instrument Co., LTD., Shanghai, China).

### 2.10. Statistical Analysis

All experiments were conducted in triplicate, and data are presented as mean ± standard deviation. The univariate analysis of variance (ANOVA) and Duncan test (significance defined as *p* < 0.05) were performed using SPSS 26 software (IBM, New York, NY, USA), and mapping was performed by employing Origin 2017 (OriginLab, Northampton, MA, USA).

## 3. Results

### 3.1. Freezing Efficiency

The freezing process of fish can generally be classified into three phases: (1) pre-cooling phase (generally from initial temperature to 0 °C); (2) phase transition (generally from 0 °C to −5 °C); and (3) deep freezing phase (generally from −5 °C to −18 °C). During the pre-cooling phase, the significant temperature difference between the sample and environment leads to a rapid release of sensible heat, causing prompt cooling of the fish sample [26]. The phase transition phase is referred to as the zone of maximum ice crystal formation, which involves ice crystal nucleation and growth. The prolonged phase transition period results in larger ice crystals and increased damage to the muscle cells [3]. As the fish reaches the freezing point, the temperature continues to drop, leading to supercooling. The fish functioning as a non-pure water system undergoes uneven nucleation driven by supercooling, with water molecules aligning on the ice core surface to form ice crystals. The conversion of free water into ice crystals results in a significant release of latent heat [27]. The frozen portion of fish in contact with the surroundings releases latent heat, causing a temperature increase. This combined effect of cooling and heat release leads to the emergence of plateau temperature, accompanied by a state of solid–liquid coexistence within the fish tissue. This plateau temperature represents the freezing point of largemouth bass, with the majority of heat being released during phase transition. During the deep-freezing phase, heat continues to be released from the cooling frozen portion, along with the release of latent heat due to the freezing of the unfrozen portion.

**Figure 1:**
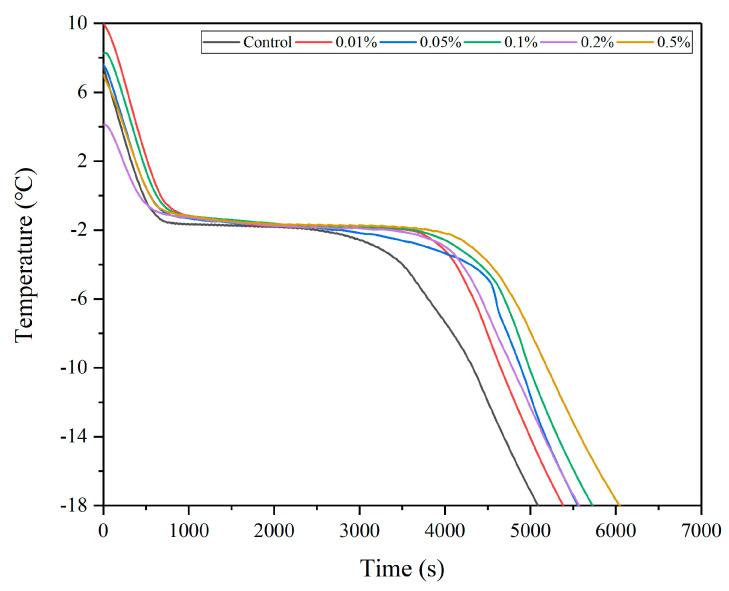
Temperature–time curves of largemouth bass treated with different concentrations (0.01%, 0.05%, 0.1%, 0.2%, 0.5%) of LeIBP during the freezing process. The results are expressed as the mean ± standard deviation (*n* = 3).

The temperature–time curve of largemouth bass treated with various concentrations of LeIBP during the freezing process is illustrated in Figure 1. The freezing point of largemouth bass in the control group was recorded as −1.69 °C. However, the freezing points of LeIBP treatment groups were significantly reduced (*p* < 0.05) to −1.82 °C, −1.82 °C, −1.85 °C, −1.88 °C, and −1.74 °C compared to the control group. Notably, the freezing point of the samples was initially decreased and then increased with an increase in LeIBP concentrations, ultimately reaching its nadir in the 0.2% LeIBP group. The data presented in Table 1 reveal that the inclusion of LeIBP exhibited a noteworthy impact on the duration required to traverse the precooling phase during the freezing process (*p* < 0.05). However, LeIBP treatment exhibited an insignificant impact on the time taken for a transition during the phase transition and deep-freezing stages (*p* > 0.05). Consequently, LeIBP significantly contributed towards the depression of the freezing point, but exhibited no impact on phase transition time. This, in turn, resulted in the formation of smaller ice crystals in the treatment groups and was responsible for preserving the structural integrity of fish cells.

### 3.2. Ice Crystal Morphology Analysis

**Figure 2:**
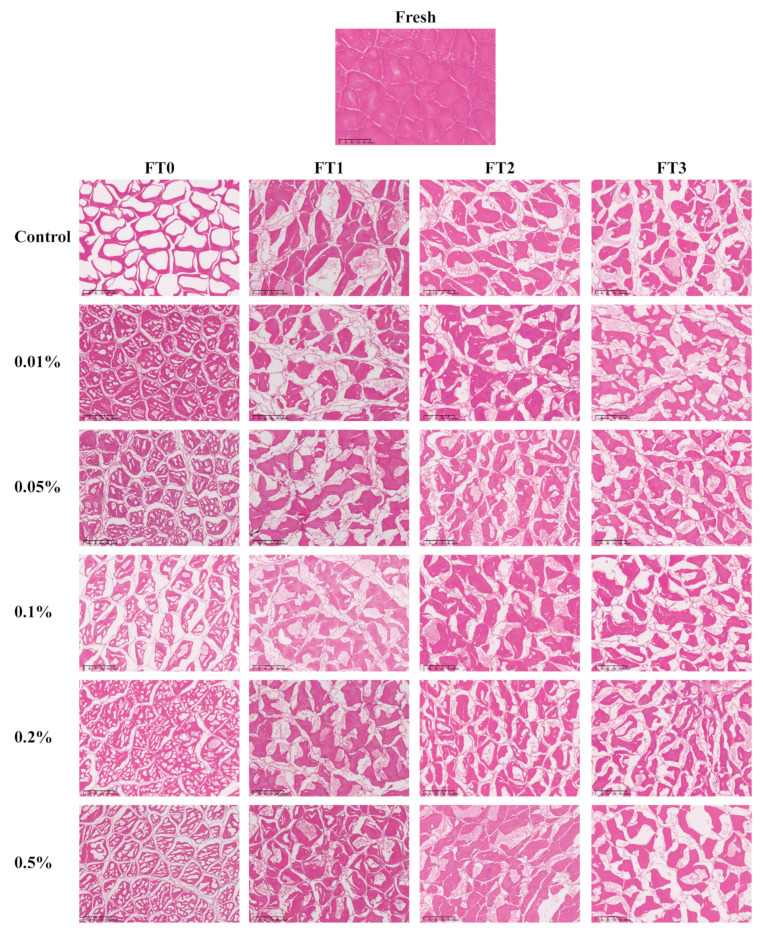
The microstructure of largemouth bass treated with different concentrations (0.01%, 0.05%, 0.1%, 0.2%, 0.5%) of LeIBP during multiple freeze–thaw cycles.

The dimensions, quantity, and location of ice crystals exhibit an immediate impact on the overall quality of frozen fish [28]. Hence, it is imperative to investigate the influence of LeIBP treatment on the morphology of ice crystals. Figure 2 illustrates the microscopic depiction of ice crystal formation within the transverse section of the muscular tissue of largemouth bass subjected to LeIBP treatment at different concentrations followed by multiple freeze–thaw cycles. The violet region in Figure 2 represents the muscular fiber of fish, while the white region signifies the presence of ice crystals.

**Figure 3:**
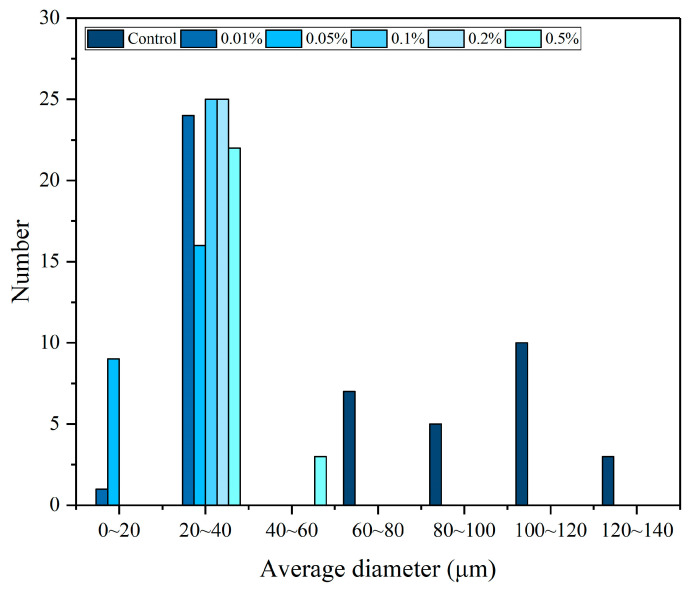
The average diameter distribution of ice crystals in largemouth bass treated with different concentrations (0.01%, 0.05%, 0.1%, 0.2%, 0.5%) of LeIBP after the first freezing.

It was observed that the fresh largemouth bass fish exhibited dense bundles of fiber with small interstitial spaces. As depicted in Table 2, after the initial freezing process, the ice crystal cross-sectional area and average diameter in the control group were 8589.46 μm^2^ and 98.47 μm, respectively. The ice crystal cross-sectional area and average diameter of samples in all treatment groups were significantly lower compared to the control group (*p* < 0.05). The lowest ice crystal cross-sectional area (347.49 μm^2^) and average diameter (21.16 μm) were observed in the 0.05% LeIBP group. Figure 3 illustrates the distribution of average crystal diameters of largemouth bass ice crystals treated with various LeIBP concentrations. The average ice crystal diameter in the control group was predominantly observed within the range of 100 to 120 μm. However, all treatment groups exhibited an average ice crystal diameter ranging from 20 to 40 μm. Thus, the addition of LeIBP notably decreased the average diameter of ice crystals. At 0.05% LeIBP, the inhibition of ice crystal growth was most effective, thus establishing this concentration as an optimal addition.

As shown in Table 2, the roundness value of the control group approaches 1, which represents an almost circular shape of ice crystals. The interplay between AFPs and the surface of ice crystals resulted in increased curvature of partial ice crystal surfaces, which in turn altered the morphology and dimensions of ice crystals, along with impeding their growth and recrystallization [29]. Simultaneously, the interaction between AFPs and the ice crystal surface induced certain damage to the ice crystal, allowing water molecules to permeate into the lattice of the crystal. It was also mentioned previously that AFPs with greater antifreeze efficacy resulted in intact ice crystal surfaces and more stable binding [10]. The elongation of ice crystals in the treatment groups (except 0.2% LeIBP group) significantly (*p* < 0.05) surpassed the elongation recorded in the control group. This observation is attributed to the thermal hysteresis activity of AFPs, which alters the growth pattern of ice crystals. When AFPs are not added, a decrease in sample temperature is typically accompanied by ice crystal formation and subsequent crystallization. Typically, ice crystals grow along the a-axis from the freezing point, resulting in the formation of hexagonal or circular ice crystals. However, with the addition of AFPs, crystallization was delayed due to the interaction of ice crystals with AFPs, although the influence of AFPs could be surmounted as the temperature decreased to a specific threshold. At this juncture, ice crystals propagated along the c-axis to form double-cone ice crystals [30].

### 3.3. Protein Secondary Structure

Raman spectroscopy is a powerful tool for analyzing alterations in the secondary structure of proteins due to its high sensitivity to molecular vibrations and minimal interference from water solvents. The secondary structure of proteins can be elucidated by examining the amide I band within the spectral range of 1600–1700 cm^−1^. This band arises from the stretching vibration of C–N and C = O bonds [25]. The secondary structure of proteins comprises four main elements, namely, α-helix (1650–1660 cm^−1^), β-sheet (1665–1680 cm^−1^), β-turn (1680 cm^−1^), and random coil (1660–1665 cm^−1^) [31]. The α-helix and β-sheet regions indicate the stability of the protein’s secondary structure, whereas the β-turn and random coil regions signify the looseness of the secondary structure. The higher proportion of α-helix signifies greater stability of the secondary structure, whereas increased β-turn and random coil content suggest a looser structure [32].

**Figure 4:**
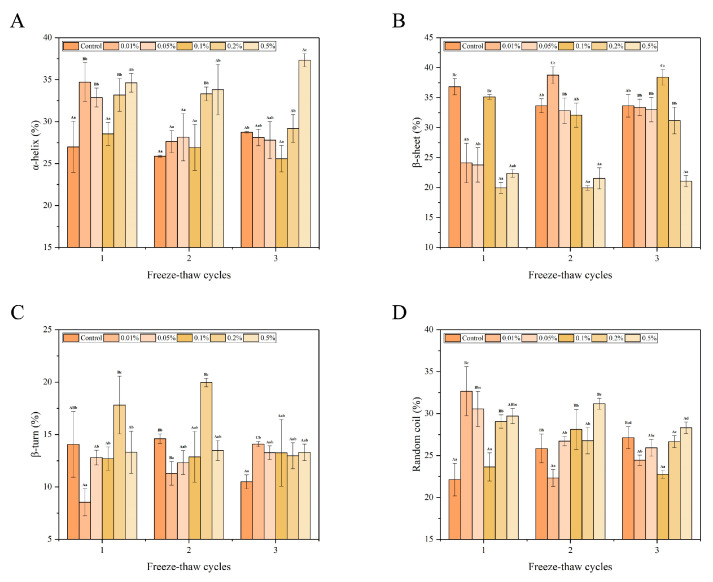
The changes in α-helix (**A**), β-sheet (**B**), β-turn (**C**), and random coil (**D**) of largemouth bass treated with different concentrations (0.01%, 0.05%, 0.1%, 0.2%, 0.5%) of LeIBP during multiple freeze–thaw cycles. The results are expressed as the mean ± standard deviation (*n* = 3). The letters (a–d) indicate significant differences (*p* < 0.05) between samples treated with different concentrations of LeIBP during the same freeze–thaw cycle, and letters (A–C) indicate significant differences (*p* < 0.05) between samples treated with the same concentration of LeIBP in different freeze–thaw cycles.

As illustrated in Figure 4, throughout the freeze–thaw cycles, except for the 0.01% and 0.2% LeIBP group, the α-helix content and β-turn content of the remaining groups exhibited no significant variation (*p* > 0.05). As the number of freeze–thaw cycles increased, the α-helix content in the 0.01% LeIBP group was decreased initially and then increased. However, the trajectory of the α-helix content in the 0.2% LeIBP group was converse. By the end of third freeze–thaw cycle, the α-helix content in both groups was decreased by 6.60% and 4.00%, respectively. The α-helix content in the 0.2% and 0.5% LeIBP group was notably higher (*p* < 0.05) during the first and second freeze–thaw cycles compared to the control group. Furthermore, significant variations (*p* < 0.05) were observed in the β-sheet content of all treatment groups (except the control and 0.5% LeIBP group) during different freeze–thaw cycles. The β-sheet content in the 0.01% LeIBP group increased initially and then decreased with an increase in the number of freeze–thaw cycles. However, the trajectory of the β-sheet content in the 0.1% LeIBP group was converse. The β-sheet content in the 0.05% and 0.2% LeIBP group increased continuously after the third freeze–thaw cycle was increased by 9.22% and 11.23%, respectively, compared to the β-sheet content observed after the initial freeze–thaw cycle. This phenomenon is attributed to the formation of smaller ice crystals during freezing in the treatment groups, which resulted in reduced cell damage and decelerated protein degradation.

As the number of freeze–thaw cycles increased, the β-turn content in the 0.01% LeIBP group increased from 8.54% to 14.08%, resulting in a 5.54% increase, while the β-turn content in the 0.2% LeIBP group increased initially and then decreased. By the end of third freeze–thaw cycle, the β-turn content in the 0.2% LeIBP group was notably lower (*p* < 0.05) compared to the first cycle. During whole freeze–thaw cycles, the random coil contents of all treatment groups (except the 0.2% LeIBP group) exhibited significant variation (*p* < 0.05). The random coil content in the control group was increased from 22.13% to 27.13% as the number of freeze–thaw cycles increased, resulting in a 5.00% increase. This could be due to the formation of large ice crystals in this group, leading to cell structure damage and the release of pro-oxidants such as lysosomal enzymes and heme iron, resulting in protein oxidation [33]. The formation and recrystallization of large intracellular ice crystals during freeze–thaw cycles may also release water, causing an increase in the intracellular ion concentration and subsequent protein denaturation [33]. As the number of freeze–thaw cycles was increased, the random coil content in the 0.05% LeIBP group decreased from 30.54% to 25.93%, showing a reduction of 4.61%, whereas the random coil content in the 0.01% LeIBP group was initially decreased and then increased. The random coil content in the 0.01% LeIBP group was notably higher (*p* < 0.05) at the end of third freeze–thaw cycle compared to the first cycle, whereas the trajectory of the random coil content in the 0.1% and 0.5% LeIBP group was converse. Overall, the stability of the protein’s secondary structure was maintained and protein denaturation was inhibited with increased content of α-helix, and the most pronounced effect was observed at 0.5% LeIBP.

### 3.4. Water-Holding Capacity

**Figure 5:**
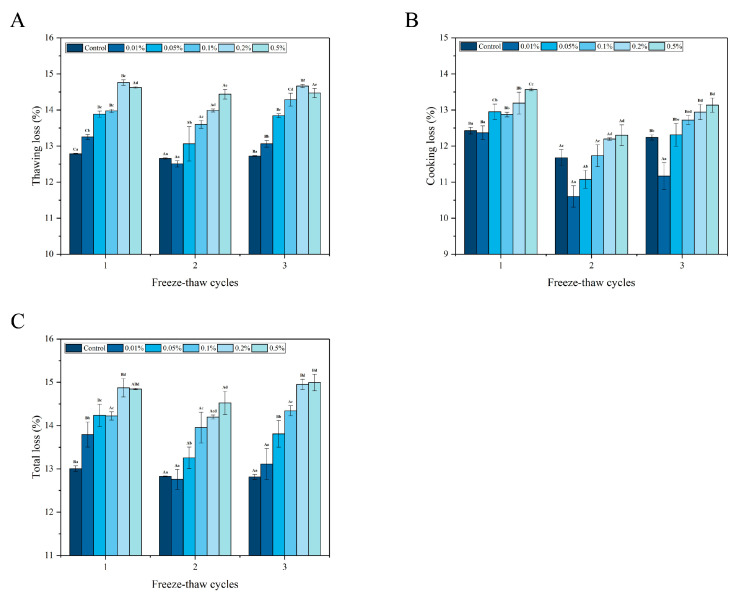
Thawing loss (**A**), cooking loss (**B**), and total loss (**C**) of largemouth bass treated with different concentrations (0.01%, 0.05%, 0.1%, 0.2%, 0.5%) of LeIBP during multiple freeze–thaw cycles. The results are expressed as the mean ± standard deviation (*n* = 3). The letters (a–f) indicate significant differences (*p* < 0.05) between samples treated with different concentrations of LeIBP during the same freeze–thaw cycle, and letters (A–C) indicate significant differences (*p* < 0.05) between samples treated with the same concentration of LeIBP in different freeze–thaw cycles.

Water-holding capacity, a pivotal metric for gauging the quality of a product, is intricately linked to sensory elements such as the juiciness, texture, and flavor of a food product [34]. Figure 5 delineates the impact of various concentrations of LeIBP treatment on the water-holding capacity of largemouth black bass during different freeze–thaw cycles.

Throughout the freeze–thaw cycles, except for the 0.5% LeIBP group, the thawing loss of the remaining groups exhibited significant variation (*p* < 0.05). Initially, the thawing loss was reduced after the second freeze–thaw cycle and then increased after the third freeze–thaw cycle. During freezing in the air, extracellular ice crystals were formed in the fish, causing water migration from the cells’ interior to the exterior due to its high chemical potential, consequently resulting in cell dehydration and osmotic pressure damage to cells [7]. Simultaneously, substantial ice crystal formation during the freezing phase of the sample caused significant damage to fish cells and muscular tissue, which in turn resulted in substantial water loss during thawing [25]. In addition, during freeze–thaw cycles, intracellular ice crystals were reduced while extracellular ice crystals were increased, and these ice crystals translated into water loss during the thawing process, thereby triggering an escalation in the thawing loss. The thawing loss during the first and third freeze–thaw cycles surged initially and then subsided with the increased concentration of LeIBP. The highest thawing loss was observed in fish samples in the 0.2% LeIBP group. This phenomenon could be attributed to the formation of a protective film on the surface of fish with an increase in the LeIBP concentration, which in turn mitigated the thawing loss. However, the trajectory of thawing loss in the second freeze–thaw cycle was converse. When the LeIBP concentration was 0.01%, the thawing loss was lowest (12.51%) compared to the control and other treatment groups. After the third freezing–thawing cycle, the thawing loss of the 0.1% LeIBP group was increased by 0.31% compared to the thawing loss observed after the first cycle, whereas the thawing losses of the other groups were reduced after the third cycle compared to the first freeze–thawing cycle. This could be ascribed to the fact that the size of the ice crystals in the 0.1% LeIBP group was the largest among all the treatment groups, which in turn disrupted cell structure and prompted the efflux of cellular fluid.

Furthermore, significant variations (*p* < 0.05) were observed in the cooking loss of all treatment groups during different freeze–thaw cycles. The cooking loss of all treatment groups decreased initially and then increased again, ultimately reaching its lowest after the second freeze–thaw cycle. Overall, after the second and third freeze–thaw cycles, the cooking loss of all treatment groups was lower compared to the initial cycle. Moreover, with an increase in LeIBP concentration, the cooking loss of all treatment groups decreased initially and then increased. The lowest cooking loss was achieved in the case of the 0.01% LeIBP group. By the end of the third freeze–thaw cycle, the cooking loss of all treatment groups, namely, the control group, 0.01%, 0.05%, 0.1%, 0.2%, and 0.5% LeIBP groups, were decreased by 0.19%, 1.20%, 0.64%, 0.16%, 0.24%, and 0.43%, respectively, compared to the first freeze–thaw cycle. The observed cooking loss was attributed to thermal denaturation of proteins, structural damage to muscles, and the loss of liquid and water-soluble nutrients during the cooking process [3]. The release of chemically bound water was linked to fat melting and protein denaturation, which led to reduced water-holding capacity of the fish [35]. The incorporation of LeIBP was found to mitigate cooking loss, with the most effective results observed in the 0.01% LeIBP group.

The total loss in the control group was reduced from 13.01% to 12.81% as the number of freeze–thaw cycles increased, resulting in a 0.20% decrease. The total loss in the 0.01%, 0.05%, and 0.2% LeIBP group presented significant variations (*p* < 0.05), with an initial decrease and then an increase with the increase in the number of freeze–thaw cycles. The total loss in the 0.01% and 0.05% LeIBP groups after the third freeze–thaw cycle decreased by 0.69% and 0.43%, respectively, compared to the total loss observed after the initial freeze–thaw cycle, whereas the total loss in the 0.2% LeIBP group was increased by 0.08%. Overall, the water-holding capacity of the fish was effectively retained at an LeIBP concentration of 0.01%.

### 3.5. Texture

The texture of fish is a reflection of its freshness and directly influences the acceptance and satisfaction of consumers [28]. As illustrated in Table 3, there was no significant difference (*p* > 0.05) in the hardness value of fish in the control group and the 0.01% LeIBP group during all three freeze–thaw cycles. The hardness of the 0.05%, 0.1%, and 0.2% LeIBP groups decreased with the progression of the freeze–thaw cycles, while the hardness of the 0.5% LeIBP group decreased initially and then increased. After the third freeze–thaw cycle, the hardness of the 0.05%, 0.1%, 0.2%, and 0.5% LeIBP groups was significantly lower (*p* < 0.05) compared to the hardness observed after the first freeze–thaw cycle. This observation was attributed to the muscle tissue damage and protein denaturation caused by ice crystals during multiple freeze–thaw cycles [29]. Within the same freeze–thaw cycle, the incorporation of LeIBP was able to enhance the hardness of fish and partially impede the quality decline. In case of springiness, no significant difference (*p* > 0.05) was observed between the control group and 0.1% LeIBP group. The springiness value of the 0.05% LeIBP group was increased with an increase in the number of freeze–thaw cycles, and the springiness value was markedly higher at the end of the third freeze–thaw cycle compared to the initial freeze–thaw cycle (*p* < 0.05). This observation was attributed to the formation of the smallest ice crystals, inflicting minimal damage to muscle tissue, thus resulting in retaining the springiness of the fish. The springiness values of the 0.01% and 0.2% LeIBP groups initially declined and then increased after the third freeze–thaw cycle, while the springiness value of the 0.5% LeIBP group decreased from 8.04 to 6.55 mm. The variations in springiness values of the fish samples may be associated with the reduction in water-holding capacity.

The viscosity of the control, 0.1%, and 0.2% LeIBP groups presented no significant differences (*p* > 0.05) during multiple freeze–thaw cycles. However, the viscosity of the 0.01% and 0.5% LeIBP groups decreased initially and then increased with the increase in number of freeze–thaw cycles. After the third freeze–thaw cycle, the viscosity of the 0.01% and 0.5% LeIBP groups was notably lower than the viscosity observed after the first freeze–thaw cycle. Additionally, the viscosity of the 0.05% LeIBP group was significantly decreased (*p* < 0.05) from 1.40 to 0.43 mJ. Regarding cohesiveness, no significant difference (*p* > 0.05) was observed in the 0.1% and 0.5% LeIBP groups, whereas the cohesiveness of the control and 0.05% LeIBP groups initially increased and then decreased with the increase in the freeze–thaw cycles. Conversely, the cohesiveness of the 0.2% LeIBP group displayed the reverse trend, while the cohesiveness of the 0.01% LeIBP group increased continuously. This phenomenon could be attributed to the formation of ice crystals, diminished intercellular binding force, and compromised muscle tissue integrity, which in turn resulted in the loss of the original firmness of the fish. However, LeIBP treatment proved to be an efficient strategy to preserve the integrity of the cell structure by inhibiting the growth and recrystallization of ice crystals in fish.

There was no significant variation (*p* > 0.05) was observed in the gumminess value of control, 0.01%, or 0.1% LeIBP groups. As the number of freeze–thaw cycles increased, the gumminess value of the 0.05% LeIBP group initially decreased and then increased, while the gumminess of the 0.2% and 0.5% LeIBP groups decreased continuously with the increase in freeze–thaw cycles. After the third freeze–thaw cycle, the gumminess of the 0.05%, 0.2%, and 0.5% LeIBP groups was notably lower compared to the first freeze–thaw cycle (*p* < 0.05). Regarding chewiness, no significant variation (*p* > 0.05) was observed in the control group, 0.05%, and 0.1% LeIBP groups during different freeze–thaw cycles. With the increase in freeze–thaw cycles, the chewiness of the 0.01% and 0.2% LeIBP groups decreased initially and then increased, while the chewiness of the 0.5% LeIBP group steadily decreased from 6.30 to 4.03 mJ. At the end of third freeze–thaw cycle, the chewiness of the 0.01%, 0.2%, and 0.5% LeIBP groups was significantly lower compared to the chewiness value observed after the first freeze–thaw cycle (*p* < 0.05). These findings suggest that the addition of LeIBP could reduce the energy required to masticate fish before swallowing after freeze–thawing. Overall, LeIBP addition impedes the decline in the gumminess and chewiness of fish samples during freeze–thaw cycles up to a certain extent.

### 3.6. Color

The color variation also serves as an indicator of meat quality and affects the market of a particular product by directly influencing the consumers’ perception and purchasing behavior. As illustrated in Table 4, after the third freeze–thaw cycle, the L* value of all treatment groups (except the 0.01% LeIBP group) was significantly increased compared to the L* value observed after the initial freeze–thaw cycle. Additionally, the L* value of all treatment groups surpassed the L* value of the control group (*p* < 0.05). As the number of freeze–thaw cycles increased, the variations in W value and L* value were similar in all treatment groups. The loss of moisture due to cell disruption and ice crystal dissolution resulted in higher brightness and whiteness of the samples, while myoglobin browning under the influence of endogenous enzymes and oxygen led to a lighter hue in fish samples [36]. Simultaneously, the moisture generated due to thawing loss formed a thin layer of water on the surface of the fish that enhanced light reflection and refraction, which in turn elevated brightness levels [25]. According to the water-holding capacity results, the inclusion of LeIBP contributed towards an increased thawing loss, resulting in an increased surface moisture of fish that contributed towards altered surface reflectivity and ultimately resulted in increased brightness and whiteness.

With an increase in the number of freeze–thaw cycles, the a* value of the control group and 0.05% LeIBP group increased continuously, ultimately reaching −2.98. Conversely, the a* value of the 0.01% LeIBP group decreased from −3.22 to −3.58, whereas the a* value of the 0.1% LeIBP group initially increased and then decreased, reaching a peak value of −3.42 after the second freeze–thaw cycle. Notably, there was no substantial difference (*p* > 0.05) observed in the a* value of the 0.2% and 0.5% LeIBP groups among them even after different freeze–thaw cycles. The myoglobin denaturation and formation of metmyoglobin during the freeze–thaw cycles were mentioned to be responsible for the decrease in a* values [37]. Furthermore, exposure of the samples to oxygen led to the conversion of myoglobin to oxygenated hemoglobin, which in turn resulted in enhanced a* values [38].

During the entirety of the freeze–thaw cycles, no significant variations (*p* > 0.05) were observed in the b* value of the control group. However, the b* values of all treatment groups initially increased and then decreased with the increase in freeze–thaw cycles. The highest b* value was observed after the second freeze–thaw cycle. After the third freeze–thaw cycle, the b* values of all treatment groups were higher compared to the b* value observed after the first freeze–thaw cycle. After the third freeze–thaw cycle, the b* value of the 0.1% LeIBP group was the highest (3.51). This phenomenon may be attributed to the fact that the ice crystals in this group were the largest among all treatment groups after initial freezing, leading to cell structure disruption, increasing fish oxidation, and ultimately resulting in an increased value of b*.

### 3.7. pH

**Figure 6:**
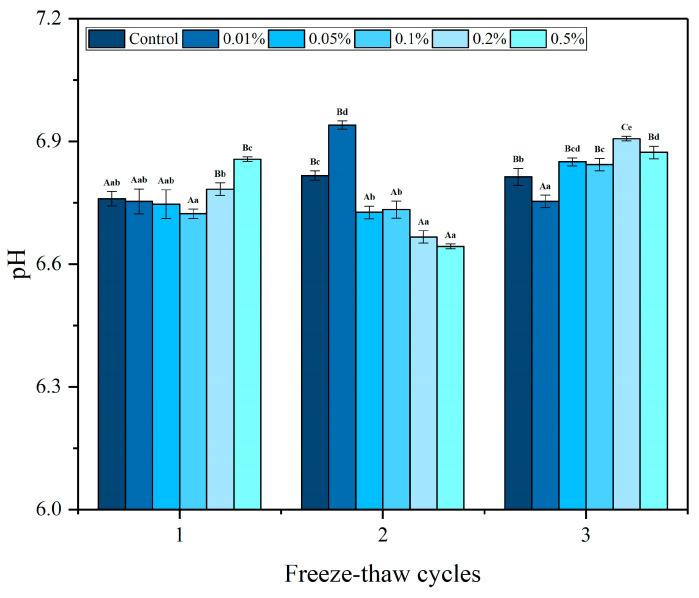
Changes in pH value of largemouth bass treated with different concentrations (0.01%, 0.05%, 0.1%, 0.2%, 0.5%) of LeIBP during multiple freeze–thaw cycles. The results are expressed as the mean ± standard deviation (*n* = 3). The letters (a–e) indicate significant differences (*p* < 0.05) between samples treated with different concentrations of LeIBP during the same freeze–thaw cycle, and letters (A–C) indicate significant differences (*p* < 0.05) between samples treated with the same concentration of LeIBP in different freeze–thaw cycles.

The pH value can be employed as an effective indicator of fish freshness. Figure 6 depicts the pH fluctuations in largemouth bass treated with different concentrations of LeIBP followed by multiple freeze–thaw cycles. Throughout the freeze–thaw process, notable and statistically significant alterations (*p* < 0.05) in the pH values of all treatment groups were observed. As the number of freeze–thaw cycles increased, the pH values of the control and 0.01% LeIBP groups increased to 6.82 and 6.94 during the second freeze–thaw cycle, respectively, and then decreased during the third freeze–thaw cycle. Conversely, the pH values of the 0.05%, 0.2%, and 0.5% LeIBP groups decreased to 6.73, 6.67, and 6.64 after the second freeze–thaw cycle, respectively. Meanwhile, the pH value of samples in the 0.1% LeIBP group increased from 6.72 to 6.84. This could be attributed to the breakdown of fish proteins into alkaline substances which, in turn, enhanced the pH of the fish. The decomposition of neutral fatty acids and phospholipase resulted in the production of free fatty acids and a subsequent decrease in pH. Furthermore, pH variations were also linked to the release of inorganic phosphate and ammonia, along with the enzymatic hydrolysis of ATP [39]. The loss of fluid from fish samples due to freezing–thawing causes an increase in solute concentration, which leads to a decrease in the pH value of a sample [33]. The release of hydrogen ions due to protein denaturation also resulted in a pH drop [40]. The initial pH level of fresh largemouth bass was recorded as 6.95. After the third freeze–thaw cycle, except the 0.01% LeIBP group, the pH values of fish samples from the other treatment groups closely approached the pH value of fresh fish, surpassing the pH value of the control group, with pH 6.91 in case of the 0.2% LeIBP group. Overall, LeIBP treatment proved to be an effective method of retaining the pH of fish along with retaining its freshness, with optimal results observed in the 0.2% LeIBP group.

## 4. Conclusions

In this study, different concentrations of LeIBP were utilized in conjunction with vacuum impregnation to process largemouth bass in order to examine the impact of LeIBP on the physicochemical attributes and microstructure of largemouth bass during freeze–thaw cycles. The LeIBP treatment significantly reduced the freezing point of largemouth bass, while no significant impact was observed on the time duration required by largemouth bass to undergo the phase transition during freezing. The control group exhibited the largest cross-sectional area and mean diameter of ice crystals, whereas a substantial decrease in both parameters was observed after LeIBP treatment. Notably, the smallest size of ice crystals was observed in the 0.05% LeIBP group. The roundness of the control group was approaching 1, whereas its elongation was noticeably lower compared to the treatment groups. After LeIBP treatment, the α-helix and random coil content of the fish sample were increased, whereas the β-sheet and β-turn content were decreased. Notably, the best inhibitory effect on protein denaturation was observed in the 0.5% LeIBP group. LeIBP addition demonstrated the capability to impede the reduction in fish water retention, with the most favorable outcome observed in the 0.01% LeIBP group. Moreover, LeIBP treatment helped to retain fish texture to a certain extent, resulting in higher L* and W values and lower a* and b* values. After third freeze–thaw cycle, the LeIBP-treated groups presented pH values similar to fresh fish. In particular, 0.05% LeIBP was regarded as an effective treatment concentration to uphold the quality of fish. Overall, this research offers constructive technical and theoretical insights for the industrial application of LeIBP to maintain the overall quality of largemouth bass during frozen storage.

## Figures and Tables

**Table 1:** The effects of different concentrations (0.01%, 0.05%, 0.1%, 0.2%, 0.5%) of LeIBP treatment on the time required for largemouth bass to pass through the pre-cooling stage, phase transition stage, and deep-freezing stage during the freezing process.

LeIBP Concentration	Pre-Cooling Stage (s)	Phase Transition Stage (s)	Deep-Freezing Stage (s)
Control	470 ± 82 a	3367 ± 434 a	1209 ± 321 a
0.01%	657 ± 100 b	3679 ± 270 ab	1033 ± 168 a
0.05%	531 ± 97 ab	3899 ± 535 ab	1101 ± 264 a
0.1%	624 ± 25 b	3930 ± 299 ab	1163 ± 157 a
0.2%	409 ± 86 a	3943 ± 172 ab	1205 ± 227 a
0.5%	549 ± 36 ab	4173 ± 175 b	1307 ± 178 a

The results are expressed as mean ± standard deviation (*n* = 3). The letters (a–b) indicate significant differences (*p* < 0.05) between samples treated with different concentrations of LeIBP.

**Table 2:** Microscopic analysis of ice crystals in largemouth bass treated with different concentrations (0.01%, 0.05%, 0.1%, 0.2%, 0.5%) of LeIBP after the first freezing.

LeIBP Concentration	Area (μm^2^)	Mean Diameter (μm)	Roundness	Elongation
Control	8589.46 ± 3139.51 b	98.47 ± 19.46 d	0.73 ± 0.12 c	1.95 ± 0.67 a
0.01%	388.53 ± 38.33 a	22.44 ± 3.18 a	0.61 ± 0.18 b	2.94 ± 1.22 b
0.05%	347.49 ± 21.12 a	21.16 ± 2.39 a	0.62 ± 0.15 b	2.90 ± 1.35 b
0.1%	550.73 ± 76.45 a	27.84 ± 3.45 b	0.59 ± 0.16 ab	2.92 ± 1.47 b
0.2%	658.57 ± 62.63 a	29.28 ± 2.36 b	0.63 ± 0.12 b	2.14 ± 0.63 a
0.5%	883.40 ± 187.94 a	34.52 ± 3.73 c	0.52 ± 0.14 a	2.95 ± 0.84 b

The results are expressed as mean ± standard deviation (*n* = 25). The letters (a–d) indicate significant differences (*p* < 0.05) between samples treated with different concentrations of LeIBP.

**Table 3:** Texture changes in largemouth bass treated with different concentrations (0.01%, 0.05%, 0.1%, 0.2%, 0.5%) of LeIBP during multiple freeze–thaw cycles.

Freeze–Thaw Cycles	LeIBP Concentration	Hardness (g)	Viscosity (mJ)	Cohesiveness	Springiness (mm)	Gumminess (g)	Chewiness (mJ)
FT1	Control	364.00 ± 28.00 Bab	1.50 ± 0.26 Ab	0.19 ± 0.01 Aa	6.65 ± 0.26 Aab	76.33 ± 2.08 Aa	5.27 ± 0.31 Aab
	0.01%	354.67 ± 34.43 Aa	0.63 ± 0.12 Ba	0.22 ± 0.01 Abc	6.37 ± 0.34 Ba	76.33 ± 2.52 Aa	6.23 ± 0.59 Bb
	0.05%	370.67 ± 29.28 Bab	1.40 ± 0.26 Bb	0.21 ± 0.01 Ab	6.40 ± 0.24 Aab	94.33 ± 4.93 Bc	5.17 ± 0.93 Aa
	0.1%	385.33 ± 4.62 Cab	0.47 ± 0.12 Aa	0.23 ± 0.01 ABcd	6.83 ± 0.18 Ab	85.33 ± 1.53 Bb	5.53 ± 0.31 Aab
	0.2%	380.00 ± 20.78 Bab	0.70 ± 0.10 Aa	0.24 ± 0.01 Bd	7.75 ± 0.11 Bc	79.00 ± 2.65 Ba	7.97 ± 0.58 Bc
	0.5%	403.33 ± 4.16 Bb	1.23 ± 0.12 Bb	0.23 ± 0.02 Acd	8.04 ± 0.25 Bc	76.67 ± 1.15 Ba	6.30 ± 0.26 Bb
FT2	Control	351.33 ± 35.12 ABb	1.13 ± 0.06 Ac	0.23 ± 0.01 Bb	6.68 ± 0.33 Ab	88.00 ± 8.89 Bc	6.07 ± 0.92 Ab
	0.01%	310.00 ± 18.00 Aa	0.37 ± 0.06 Aa	0.22 ± 0.01 Ab	5.60 ± 0.21 Aa	71.67 ± 6.51 Aab	4.47 ± 0.40 Aa
	0.05%	332.67 ± 9.02 Aab	1.30 ± 0.26 Bc	0.29 ± 0.04 Bc	6.65 ± 0.17 Ab	68.33 ± 12.10 Aab	4.17 ± 1.50 Aa
	0.1%	360.00 ± 5.29 Bb	0.67 ± 0.15 Ab	0.22 ± 0.01 Ab	6.73 ± 0.60 Ab	81.33 ± 6.03 ABbc	5.23 ± 0.38 Aab
	0.2%	369.33 ± 6.43 Bb	0.63 ± 0.06 Ab	0.18 ± 0.01 Aa	5.92 ± 0.49 Aa	64.33 ± 2.89 Aa	3.70 ± 0.70 Aa
	0.5%	334.67 ± 21.39 Aab	0.47 ± 0.12 Aab	0.23 ± 0.02 Ab	6.75 ± 0.34 Ab	74.67 ± 8.50 ABabc	4.20 ± 0.56 Aa
FT3	Control	304.67 ± 15.01 Aa	1.20 ± 0.17 Ac	0.22 ± 0.01 Ba	6.88 ± 0.25 Aab	78.00 ± 2.65 ABb	5.10 ± 0.56 Ab
	0.01%	313.33 ± 5.77 Aa	0.50 ± 0.10 ABa	0.25 ± 0.01 Bd	6.85 ± 0.32 Bab	79.67 ± 7.02 Ab	5.23 ± 0.59 ABb
	0.05%	324.67 ± 1.15 Aab	0.43 ± 0.06 Aa	0.25 ± 0.01 Acd	7.23 ± 0.13 Bb	78.00 ± 1.73 Ab	4.13 ± 0.25 Aa
	0.1%	318.00 ± 8.00 Aa	0.73 ± 0.12 Ab	0.24 ± 0.01 Bbc	7.25 ± 0.17 Ab	76.67 ± 2.08 Ab	5.10 ± 0.52 Ab
	0.2%	313.33 ± 2.31 Aa	0.73 ± 0.06 Ab	0.23 ± 0.01 Bab	7.20 ± 0.21 Bb	64.00 ± 1.00 Aa	4.20 ± 0.35 Aa
	0.5%	348.67 ± 28.59 Ab	0.50 ± 0.10 Aa	0.24 ± 0.01 Abc	6.55 ± 0.23 Aa	64.67 ± 2.52 Aa	4.03 ± 0.12 Aa

The results are expressed as mean ± standard deviation (*n* = 3). The letters (a–d) indicate significant differences (*p* < 0.05) between samples treated with different concentrations of LeIBP during the same freeze–thaw cycle, and letters (A–C) indicate significant differences (*p* < 0.05) between samples treated with the same concentration of LeIBP in different freeze–thaw cycles.

**Table 4:** Color changes in largemouth bass treated with different concentrations (0.01%, 0.05%, 0.1%, 0.2%, 0.5%) of LeIBP during multiple freeze–thaw cycles.

Freeze–Thaw Cycles	LeIBP Concentration	L *	a *	b *	W
FT1	Control	37.31 ± 0.59 Aa	−3.52 ± 0.10 Ab	3.18 ± 0.01 ABc	37.13 ± 0.58 Aa
	0.01%	44.39 ± 0.76 Ad	−3.22 ± 0.10 Ba	1.99 ± 0.54 Ab	44.26 ± 0.77 ABd
	0.05%	42.36 ± 0.54 Ac	−3.65 ± 0.17 Ab	1.52 ± 0.41 Aab	42.22 ± 0.55 Ac
	0.1%	43.18 ± 0.41 Ac	−3.60 ± 0.05 Ab	1.56 ± 0.15 Aab	43.04 ± 0.42 Ac
	0.2%	42.49 ± 0.17 Ac	−3.50 ± 0.04 Ab	1.26 ± 0.25 Aa	42.37 ± 0.17 Ac
	0.5%	40.61 ± 0.31 Ab	−3.56 ± 0.05 Ab	1.54 ± 0.12 Aab	40.48 ± 0.31 Ab
FT2	Control	43.61 ± 0.41 Cab	−3.48 ± 0.11 Abc	4.06 ± 0.78 Bcd	43.35 ± 0.47 Ca
	0.01%	44.30 ± 0.65 Ab	−3.53 ± 0.11 Aabc	4.46 ± 0.41 Bd	44.01 ± 0.66 Aa
	0.05%	47.75 ± 0.33 Cd	−3.61 ± 0.07 Aab	3.68 ± 0.23 Cbc	47.50 ± 0.34 Cc
	0.1%	43.45 ± 0.61 Aa	−3.42 ± 0.08 Bc	4.28 ± 0.14 Ccd	43.19 ± 0.60 Aa
	0.2%	47.07 ± 0.22 Cd	−3.65 ± 0.13 Aab	3.32 ± 0.23 Cb	46.84 ± 0.23 Cc
	0.5%	45.87 ± 0.11 Cc	−3.66 ± 0.04 Aa	2.37 ± 0.18 Ba	45.70 ± 0.11 Cb
FT3	Control	42.38 ± 0.30 Ba	−2.98 ± 0.10 Bb	2.95 ± 0.39 Ac	42.23 ± 0.30 Ba
	0.01%	45.82 ± 0.68 Bd	−3.58 ± 0.05 Aa	2.52 ± 0.25 Abc	45.64 ± 0.69 Bd
	0.05%	44.11 ± 0.38 Bbc	−2.98 ± 0.05 Bb	2.31 ± 0.28 Bab	43.98 ± 0.37 Bbc
	0.1%	45.29 ± 0.16 Bcd	−3.62 ± 0.08 Aa	3.51 ± 0.25 Bd	45.06 ± 0.16 Bcd
	0.2%	46.37 ± 0.19 Bd	−3.63 ± 0.03 Aa	2.85 ± 0.22 Bbc	46.17 ± 0.19 Bd
	0.5%	43.74 ± 1.44 Bb	−3.51 ± 0.14 Aa	1.88 ± 0.39 ABa	43.60 ± 1.46 Bb

L *, a *, and b *, respectively, represent lightness value, redness value, and yellowness value. The results are expressed as mean ± standard deviation (*n* = 3). The letters (a–d) indicate significant differences (*p* < 0.05) between samples treated with different concentrations of LeIBP during the same freeze–thaw cycle, and letters (A–C) indicate significant differences (*p* < 0.05) between samples treated with the same concentration of LeIBP in different freeze–thaw cycles.

## Data Availability

The data presented in this study are available on request from the corresponding author due to privacy.
